# Ewing sarcoma of the thumb presenting in a Hispanic patient: A case report

**DOI:** 10.1016/j.ijscr.2024.110552

**Published:** 2024-11-06

**Authors:** Carlos Guevara, José I. Acosta Julbe, Derick Rodríguez-Reyes, Juan Bibiloni

**Affiliations:** a Department of Orthopedic Surgery, University of Puerto Rico Medical Sciences Campus, Puerto Rico; b Department of General Surgery, University of Puerto Rico Medical Sciences Campus, School of Medicine, Puerto Rico

**Keywords:** Ewing sarcoma, Metastasis, Case report, Thumb, Disarticulation, Chemotherapy

## Abstract

**Introduction:**

Ewing sarcoma (EwS) is an uncommon and highly aggressive cancer primarily affecting children and young adults. This tumor constitutes 10 % to 15 % of all bone sarcomas and often presents in the pelvis, axial skeleton, and femur. Despite its rarity, EwS's rapid progression and early metastatic potential make it a significant concern in pediatric oncology, highlighting the need for effective treatment protocols and further research.

**Case presentation:**

We report the case of an 18-year-old Hispanic male who presented with an initially asymptomatic growing mass in his right distal left thumb. The diagnosis of an extra-axial EwS was confirmed after histopathologic evaluation. He was managed with a trans-interphalangeal disarticulation followed by adjuvant chemotherapy with no signs of recurrence at 24-months. This case has been reported in line with the SURGICAL CASE Reports (SCARE) guidelines.

**Discussion:**

EwS is known for rapid growth and early metastasis, often remaining asymptomatic until advanced stages, complicating treatment and reducing survival. Common symptoms include tenderness and swelling. In our case, the patient presented with a slowly enlarging, initially asymptomatic thumb mass, leading to delayed diagnosis. EwS in the hand, especially the thumb, is rare, with only nine cases reported.

**Conclusion:**

While EwS rarely manifests in fingers, it remains crucial to include this diagnosis and other malignant tumors as a potential consideration when evaluating lesions found in this area.

## Highlights

• Ewing sarcoma rarely presents in the thumb.
• Early diagnosis in young patients with bening-appearing masses is crucial for improved outcomes.

## 1 Introduction

Ewing sarcoma (EwS) is an uncommon and highly aggressive cancer primarily affecting children and young adults, constituting 10 % to 15 % of all bone sarcomas [1]. With an incidence rate of 7.5 million annually, EwS represents the second most common bone sarcoma [2]. Nonmetastatic disease has a five-year survival rate of 75–80 %, while metastatic disease is around 30 % [1]. According to Nathan PC et al., the average cost of treating EwS in pediatric centers exceeded $200,000 compared to approximately $73,000 in adult centers, highlighting the substantial financial burden associated with their management [3].

Studies have shown that EwS typically presents in the pelvis, axial skeleton, and femur [1]. According to McBee et al., EwS of the hand is exceptionally rare, with few cases reported to date [4]. Fujii et al. found that most cases affect the proximal phalanx of the third digit [5]. In addition, only one case has been reported in a Hispanic patient [4]. Due to the rarity of EwS and similar tumors in the hand, standardized treatment protocols have not been established [6]. Nevertheless, current evidence suggests that the primary treatment approach should include chemotherapy combined with a wide local excision of the tumor [7]. There is scarce literature on Hispanic patients developing EwS of the digits and their outcomes.

We present the case of an 18-year-old Hispanic male with EwS of the left thumb managed with a disarticulation at the interphalangeal joint followed by adjuvant chemotherapy with no evidence of disease at 24 months. We obtained written consent from the patient to use his de-identified medical information.

## 2 Case presentation

This case has been reported in line with the SURGICAL CASE Reports (SCARE) criteria [8].

**Fig. 1:**
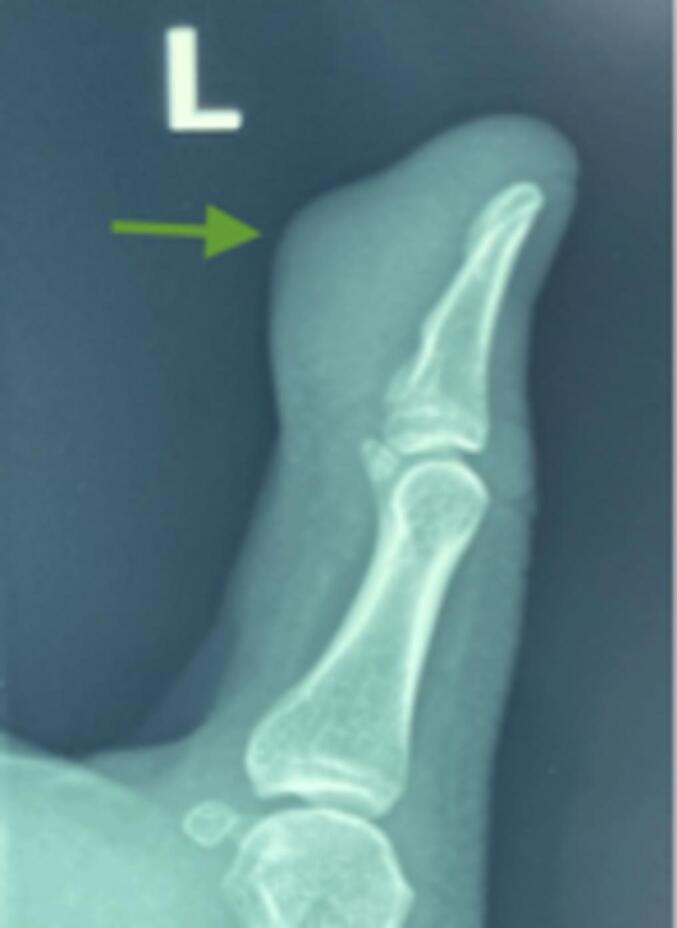
Radiographs (anteroposterior views) of the left thumb shows swelling of the soft tissue and increased density of the distal phalanx of the 5th finger (arrow).

**Fig. 2:**
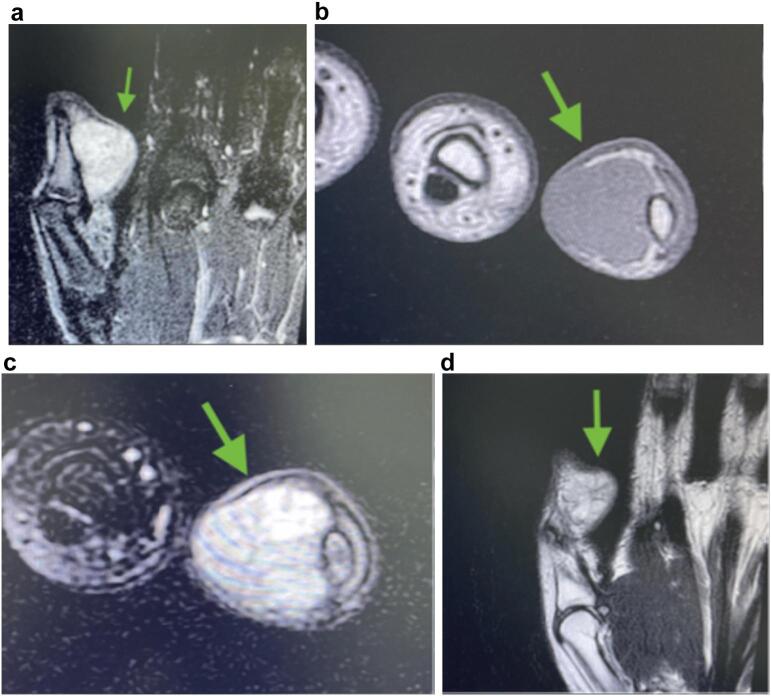
Magnetic Resonance Imaging of the left thumb [coronal view of STIR sequence (A), axial view of Contrast-enhanced T1-weighted sequence (B), axial view of STIR sequence (C) and T2-weighted sequence (D)] showing hyperintense areas on palmar side of the distal thumb.

A previously healthy 18-year-old Hispanic male presented to an orthopedic hand clinic with a complaint of a right thumb mass. He noticed a small non-tender mass in the distal left thumb seven months before his first evaluation. He did not recall any preceding trauma but was concerned that it had grown steadily and became softer and depressible. The patient denied experiencing systemic symptoms, including fever, fatigue, or weight loss. The patient remained asymptomatic, and the lesion caused minimal discomfort. However, three months later, he started experiencing worsening symptoms, including tenderness and growth of the mass, and decided to visit an orthopedic hand clinic. Radiographs of the left hand showed soft tissue swelling around the distal thumb (Fig. 1). The contrast-enhanced magnetic resonance imaging (MRI) revealed an extra-osseous heterogenous lobulated mass in the distal phalanx of the left thumb (Fig. 2). The initial interpretation suggested a giant cell tumor of the tendon sheath.

**Fig. 3:**
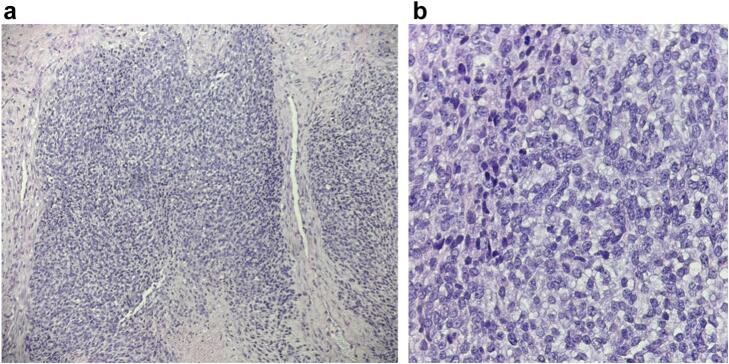
Microscopic evaluation – hematoxylin and eosin stain of surgical specimen in low power (4×) magnification (A), and high power (40×) magnification (B).

Despite the initial benign appearance and the MRI findings, the surgeon opted for an incisional biopsy through the distal phalanx, given the continual growth and tenderness. The procedure was conducted without complications, and a 3.7 × 3.2 cm mass was resected. A sample was sent to a national referral center for diagnostic confirmation, and high mitotic activity (16 mitoses per millimeters^2^ at >40/10 high power field), the atypical blue round cells, irregular nuclear contours, coarse chromatin, prominent nucleoli, and focal tumor necrosis were noted on microscopic evaluation (Fig. 3). The tissue analysis was consistent with the diagnosis of EwS. His first postoperative visit showed satisfactory recovery without immediate concerns and a properly healing wound. At this point, the patient was referred to our musculoskeletal (MSK) oncology clinic.

On his initial visit, a physical examination revealed a tender and fixed mass with an erythematous and ulcerated appearance measuring approximately 3 × 3 centimeters in the dorsal aspect of the distal left thumb (Fig. 1). Chest tomography and whole-body bone scan showed no metastatic disease. He denied allergies or malignancy and a tonsillectomy ten years before the presentation. The family history was negative for malignancies, and the social history was negative for smoking, alcohol abuse, or drug use.

Although the protocol followed at our institution includes neoadjuvant chemotherapy, we decided to recommend surgical resection as the initial treatment due to constant, intractable arterial bleeding at the tumor site, due to the tumor location eroding the digital artery. After a thorough discussion, including the risks and benefits of the procedure and his consent, the patient underwent an interphalangeal disarticulation followed by adjuvant chemotherapy. The surgical margins were negative. The chemotherapy regimen consisted of six rounds of Vincristine, Doxorubicin, Cyclophosphamide, Ifosfamide, and Etoposide (VDC-IE) chemotherapy, which started two months after the intervention. At his 24-month follow-up visit, the patient remains free of the disease and independent in his activities of daily living.

## 3 Discussion

EwS is the second most common primary bone sarcoma in children and adolescents, though its incidence in the hand and feet is reported to be <1 %, primarily affecting individuals between 15 and 20 years of age [2,9,10]. We report the case of an 18-year-old Hispanic male who developed an EwS in the tip of his left thumb. This tumor was successfully managed with a disarticulation at the interphalangeal joint followed by adjuvant chemotherapy, with no signs of recurrence or systemic disease after 24 months of follow-up.

EwS progresses rapidly, often with early metastasis, making it a concern in pediatric oncology [11,12]. It typically presents with tenderness and swelling after significant growth [1]. In our case, the patient had a slowly enlarging, asymptomatic thumb mass, leading to a delayed diagnosis. EwS in the hand is rare, with only nine cases reported in the thumb (summarized in Table 1) [13]. Unlike McBee et al.'s case in the dorsal web space [4], our case involved a mass distal to the thumb's interphalangeal joint, an uncommon location for EwS.

**Table 1:** Reported cases of Ewing sarcoma of the thumb.

Case	Author (last name)	Year of publication	Age	Sex	Location
1	Chen	1983	51	F	Proximal phalanx
2	Jones	1993	12	F	Distal phalanx
3	Yamaguchi	1997	51	M	Distal phalanx
4	Seroussi	2004	10	F	Proximal phalanx
5	Baccari	2012	10	M	Distal phalanx
6	Jamshidi	2013	28	M	Distal phalanx
7	Gowdy	2014	10	F	Proximal phalanx
8	Le Deley	2022	10	M	Proximal phalanx
9	McBee	2023	15	M	Between first and second dorsal webspace

The Hispanic ethnicity of our patient adds to the rarity of our case [14,15]. While Wiemels et al. found that Hispanics have a lower risk of EwS compared to non-Hispanic populations [16], Dhir et al. reported a higher-than-expected incidence of osseous EwS among Florida Hispanic males compared to Hispanics from other U.S. regions [17]. Additionally, Koohbanani et al. identified Hispanic ethnicity as an independent poor prognostic factor [18]. The only other report of EwS of the hand was described by McBee et al. [4], yet we are the first to describe a young Hispanic male patient presenting with EwS of the thumb.

Our patient presented to the musculoskeletal oncology clinic seven months after noticing the mass. Late presentation of soft tissue sarcomas is common [19]. Sharib et al. found that Latino patients with sarcomas often seek care in the emergency room and are diagnosed younger [15], while Brasme et al. noted delayed diagnosis of EwS, especially in adolescents and extremity cases [20]. In our opinion, the delay in this patient seeking medical attention was due to the unusual presentation and benign initial appearance. Additionally, lower insurance rates and socioeconomic status among Hispanics contribute to care delays and worse outcomes in childhood cancers [[21], [22], [23]].

MRI remains the preferred modality for diagnostic imaging due to its superior soft tissue contrast [7]. In this case, the preoperative MRI showed an extra-osseous heterogeneous lobulated mass on the distal phalanx of the left thumb. These findings were initially consistent with a giant cell tumor of the tendon sheath, which is the second most common benign neoplasm in the hand after ganglion cysts [24,25]. Although MRI has a high negative predictive value for malignant bone tumors, its findings may be nonspecific, and other benign tumors are frequently diagnosed [26].

Given the benign initial appearance and MRI findings, the team opted for a biopsy, which remains the cornerstone of diagnosis [1]. The results confirmed the diagnosis of EwS. This patient presented with persistent and intractable arterial bleeding at the tumor site, which was due to the tumor location eroding the digital artery and causing the arterial bleeding. For this reason, we decided to bypass our treatment protocol for EwS, which includes neoadjuvant chemotherapy, and recommended surgical resection as the initial treatment. Thus, the patient was managed with a disarticulation at the level of the interphalangeal joint, followed by adjuvant chemotherapy to address both the primary tumor and potential metastatic disease. Our management aligns with literature recommendations, in which surgical excision followed by advocated chemotherapy as the treatment of choice [[27], [28], [29], [30]]. Metastatic disease at presentation is the strongest predictor of survival in patients with EwS, followed by tumor necrosis because of neoadjuvant therapy [31]. Yet, Bacci et al. found that adjuvant and neoadjuvant chemotherapy resulted in comparable results in patients with localized disease [32]. In addition, the recommendations established by Werier et al. include that the local treatment for patients with EwS of the bone should be decided by a multidisciplinary tumor board together with the patient after considering the patient characteristics (i.e., tumor size, location, and existing comorbidities) [33]. Following the Euro-EWING 99 trial guidelines, our oncology team administered six rounds of VDC-IE chemotherapy two months after the intervention [34]. Froeb et al. conducted a randomized controlled trial assessing the protocols of the Cooperative Ewing Sarcoma Study Group (CESS) from 1991 to 2009 (i.e., EICESS-92 and EURO-E.W.I.N.G.99) [35]. They reported an event-free survival rate of 66 % with the combination of chemotherapy and surgery for EwS located in the hand or feet [35].

## 4 Conclusion

We report the case of an 18-year-old Hispanic male with EwS of the thumb, successfully managed with disarticulation at the interphalangeal joint and adjuvant chemotherapy, with no evidence of disease two years post-surgery. Our case underscores the importance of clinical suspicion in adolescents with benign-appearing masses in the distal phalanx or hand. It also contributes to the understanding of this disease's presentation in a diverse population, such as Hispanics.

## Author contribution

Carlos Guevara-Serra, MD: Conceptualization, investigation, data curation, and writing.

José I. Acosta Julbe, MD: Investigation, data curation, visualization, writing, and editing.

Derick Rodriguez Reyes, MD: Writing, reviewing, and editing.

Juan Bibiloni, MD: Investigation, supervision, reviewing, and editing.

## Consent

Written informed consent was obtained from the patient's parents/legal guardian for publication and any accompanying images. A copy of the written consent is available for review by the Editor-in-Chief of this journal on request.

## Ethical approval

This study is a case report and does not require ethical from our institution's IRB. The patient was not physically involved in the research and only previously collected data was used. However, we have complied with HIPPA to protect the patient's information.

## Guarantor

Carlos Guevara-Serra, MD and José I. Acosta Julbe, MD; Department of Orthopaedic Surgery, University of Puerto Rico, Medical Sciences Campus, San Juan, Puerto Rico.

## Research registration number

Not applicable. This case report does not consist of a “First in Man case report.”

## Declaration of Generative AI and AI-assisted technologies in the writing process

This work's author(s) did not use AI and AI-assisted technologies in writing.

## Funding

The authors received no financial support for this study. No relevant financial activities outside the submitted work.

## Conflict of interest statement

The authors report no conflicts of interest.

## References

[1] Durer S., Gasalberti D.P., Shaikh H. (2024).

[2] Spector L.G., Hubbard A.K., Diessner B.J., Machiela M.J., Webber B.R., Schiffman J.D. (2021). Comparative international incidence of Ewing sarcoma 1988 to 2012. Int. J. Cancer.

[3] Nathan P.C., Bremner K.E., Liu N. (2019). Resource utilization and costs in adolescents treated for cancer in pediatric vs adult institutions. J. Natl. Cancer Inst..

[4] McBee D.B., Bentley H.A., Toledanes G. (2023). Ewing’s sarcoma of the hand: an unusual presentation in a young Hispanic male. Cureus.

[5] Fujii H., Honoki K., Kobata Y., Yajima H., Kido A., Takakura Y. (2014). Ewing sarcoma of the proximal phalanx: case report. J. Plast. Surg. Hand Surg..

[6] Joseph S.A., Bhandari R., Albert A. (2018). Saving the hand: role of multimodality therapy for Ewing’s sarcoma family tumor of the palm. Adv. Radiat. Oncol..

[7] Zollner S.K., Amatruda J.F., Bauer S. (2021). Ewing sarcoma-diagnosis, treatment, clinical challenges and future perspectives. J. Clin. Med..

[8] Sohrabi C., Mathew G., Maria N. (2023). The SCARE 2023 guideline: updating consensus Surgical CAse REport (SCARE) guidelines. Int. J. Surg..

[9] Shekhar A., Korlhalli S., Murgod G. (2015). Ewing’s sarcoma of proximal phalanx of the hand with skip metastases to metacarpals. Indian J. Orthop..

[10] Khan S., Abid Z., Haider G. (2021). Incidence of Ewing’s sarcoma in different age groups, their associated features, and its correlation with primary care interval. Cureus.

[11] Wang C.C., Schulz M.D. (1953). Ewing’s sarcoma; a study of fifty cases treated at the Massachusetts General Hospital, 1930-1952 inclusive. N. Engl. J. Med..

[12] Dahlin D.C., Coventry M.B., Scanlon P.W., Ewing’s sarcoma. (1961). A critical analysis of 165 cases. J. Bone Joint Surg. Am..

[13] McManus D.P. (1990). Points in question. Genetic diversity in Echinococcus granulosus. Int. J. Parasitol..

[14] Self C., MacQuarrie K.L., Cost C.R. (2022). Osteosarcoma/Ewing sarcoma. Pediatr. Rev..

[15] Sharib J., Horvai A., Gray Hazard F.K. (2014). Comparison of Latino and non-Latino patients with Ewing sarcoma. Pediatr. Blood Cancer.

[16] Wiemels J.L., Wang R., Feng Q. (2023). Birth characteristics and risk of Ewing sarcoma. Cancer Causes Control.

[17] Dhir A., Rahul R., Liu Q. (2024). Disparities in incidence and survival for patients with Ewing sarcoma in Florida. Cancer Med..

[18] Koohbanani B., Han G., Reed D. (2013). Ethnicity and age disparities in Ewing sarcoma outcome. Fetal Pediatr. Pathol..

[19] Hayes A.J., Nixon I.F., Strauss D.C. (2024). UK guidelines for the management of soft tissue sarcomas. Br. J. Cancer.

[20] Brasme J.F., Chalumeau M., Oberlin O., Valteau-Couanet D., Gaspar N. (2014). Time to diagnosis of Ewing tumors in children and adolescents is not associated with metastasis or survival: a prospective multicenter study of 436 patients. J. Clin. Oncol..

[21] Gupta S., Wilejto M., Pole J.D., Guttmann A., Sung L. (2014). Low socioeconomic status is associated with worse survival in children with cancer: a systematic review. PLoS One.

[22] Sampagar A., Keerthana S., Dias M.C., Reddy N.A., Patil N. (2023). A study of factors influencing delayed diagnosis in pediatric cancers: a step towards better outcomes-a cross-sectional study. J. Pediatr. Hematol. Oncol..

[23] Gavidia R., Fuentes S.L., Vasquez R. (2012). Low socioeconomic status is associated with prolonged times to assessment and treatment, sepsis and infectious death in pediatric fever in El Salvador. PLoS One.

[24] Di Grazia S., Succi G., Fragetta F., Perrotta R.E. (2013). Giant cell tumor of tendon sheath: study of 64 cases and review of literature. G. Chir..

[25] Kolisetty P.V., Ali S.S., Ahmad I., Sudhy I.K., Prakash O., Kishore Y.R. (2024). Giant cell tumor of the tendon sheath of the hand: analysis of factors impacting recurrence. Indian J. Plast. Surg..

[26] Ulaner G., Hwang S., Landa J., Lefkowitz R.A., Panicek D.M. (2013). Musculoskeletal tumours and tumour-like conditions: common and avoidable pitfalls at imaging in patients with known or suspected cancer: part B: malignant mimics of benign tumours. Int. Orthop..

[27] San-Julian M., Duart J., de Rada P.D., Sierrasesumaga L. (2008). Limb salvage in Ewing’s sarcoma of the distal lower extremity. Foot Ankle Int..

[28] Seroussi D., Renauld V., Hebrard W., Duport G., Cohrs D. (2004). Ewing sarcoma of thumb: report of a case and review of the literature. Ann. Chir. Plast. Esthet..

[29] Jerome T.J., Varghese M., Sankaran B. (2008). Ewing’s sarcoma of the distal phalanx of the little finger. J. Hand Surg. Eur..

[30] Lacey S.H., Danish E.H., Thompson G.H., Joyce M.J. (1987). Ewing sarcoma of the proximal phalanx of a finger. A case report. J. Bone Joint Surg. Am..

[31] Rebets Y., Kormanec J., Lutzhetskyy A., Bernaerts K., Anne J. (2023). Cloning and expression of metagenomic DNA in Streptomyces lividans and its subsequent fermentation for optimized production. Methods Mol. Biol..

[32] Bacci G., Balladelli A., Forni C. (2007). Adjuvant and neoadjuvant chemotherapy for Ewing sarcoma family tumors in patients aged between 40 and 60: report of 35 cases and comparison of results with 586 younger patients treated with the same protocols in the same years. Cancer.

[33] Werier J., Yao X., Caudrelier J.M. (2016). Evidence-based guideline recommendations on treatment strategies for localized Ewing’s sarcoma of bone following neo-adjuvant chemotherapy. Surg. Oncol..

[34] Anderton J., Moroz V., Marec-Berard P. (2020). International randomised controlled trial for the treatment of newly diagnosed EWING sarcoma family of tumours - EURO EWING 2012 Protocol. Trials.

[35] Froeb D., Ranft A., Boelling T. (2012). Ewing sarcoma of the hand or foot. Klin. Padiatr..

